# Resistant Bacteria in Broiler Litter Used as Ruminant Feed: Effect of Biotic Treatment

**DOI:** 10.3390/antibiotics12071093

**Published:** 2023-06-23

**Authors:** Solomon Efriem, Chris Sabastian, Shlomo Blum, Marcelo Fleker, Sameer J. Mabjeesh, Malka Britzi

**Affiliations:** 1 The Robert H. Smith Faculty of Agriculture, Food and Environment, Hebrew University, Rehovot 7610001, Israel; solefriem@gmail.com (S.E.);; 2 National Residue Control Laboratory, Kimron Veterinary Institute, Beit Dagan 5025001, Israel; malkabr@gmail.com; 3 Bacteriology and Mycology Laboratory, Kimron Veterinary Institute, Beit Dagan 5025001, Israel

**Keywords:** antimicrobial resistance, antimicrobials, coccidiostats, MALDI-TOF-MS analysis, Kirby–Bauer test, broiler litter

## Abstract

The use of antimicrobial drugs and coccidiostats in poultry farming is widespread, with a significant proportion of these drugs being excreted and released into the environment. The residues of such drugs in poultry litter (PL) can result in the development of antibiotic-resistant bacteria. The impact of different biotic treatments (aerobic, anaerobic, and stacking) on broiler litter (BL) before its use as animal feed has not been studied extensively, nor have the differences between antimicrobial-dependent and independent broiler farms been investigated. This study aimed to determine the resistant bacteria in BL used as ruminant feed before and after litter treatment. The results show that the most resistant bacteria before BL treatment were the Enterococcus species. This study also found that the quantity of amoxicillin-resistant *Enterococcus* detected in samples from antimicrobial-dependent farms was significantly higher than in those from antimicrobial-independent farms. Additionally, 14% of bacteria were multi-resistant to tetracycline, sulfafurazole, and erythromycin in antimicrobial-independent farm litters, significantly lower than those measured in antimicrobial-dependent broiler farm litter. This study highlights the importance of better understanding, regulating, managing, and using animal waste appropriately to reduce the number of antibiotic-resistant bacteria and minimize the use of antimicrobials that carry high risks for animals, humans, and the environment

## 1. Introduction

Poultry farms are the largest livestock farms and use increasing amounts of antimicrobials and coccidiostats; therefore, they have more influence on spreading selective antimicrobial-resistant bacteria in the environment. Broiler chicken-producing operations (CAFO) systems and the selective breeding of broilers that grow faster than the previous generation, as well as the crowded factory farming milieu, have effects on the chicken immune system [1]. Chickens are grown on organic bedding and thus ingest their litter [2]. All these reasons force the use of antimicrobials and coccidiostats in broiler-growing systems. The use of a low minimum inhibitory concentration (MIC) and residue concentrations of drugs can lead to the development of selective resistant bacteria in the litter. According to EU council directive 2007/43/EC, reducing chicken stocking density (from 39 to 33 Kg/m^3^) was not effective to reduce the use of antimicrobials [3,4]. However, slower-growing chickens with low stock density reduce the use of antimicrobial compared to fast-growing chickens [5]. The organic and slower-growing breed farming can reduce the use of antimicrobial but they are more expensive than conventional broiler systems.

According to Witte, the widespread use of antimicrobials that select for communities of resistant bacteria in litter is one of the problems of global production [6]. The pharmacokinetics of antimicrobials in litter decrease the profile of bacterial susceptibility, such that resistant bacteria grow easily. Animal manure, e.g., BL spreads the resistant bacteria to the environment via selective pressure, spontaneous mutation and the unreasonable use of antimicrobials [7,8]. One of the routes through which antimicrobial-resistant bacteria are spread to the environment is via the food animal production chain, like poultry, pork and beef. The presence of residue antimicrobials in the environment changes the soil structure and influence on degradation and microorganism diversity [9]. The use of antimicrobials and their influences on selection antimicrobial bacteria have also been studied in depth [10]. The residues of sulfadiazine and oxytetracycline at low MIC were effective in causing selective pressure on the resistant population [11]. However, the threshold concentration of antimicrobials has still not been sufficiently researched. The residue antimicrobials in low concentration < MIC and co-selection compounds like coccidiostats require more study to calculate the selection pressure concentration of residue drugs on resistant bacteria and their chronic and acute ecotoxicity effects on the environment.

The WHO recognized multi-antimicrobial resistance as one of top 10 global public health threats due the environment contamination caused by resistant bacteria. The nosocomial pathogenic ESKAPE (*Enterococcus faecium*, *Staphylococcus aureus*, *Klebsiella pneumoniae*, *Acinetobacter baumannii*, *Pseudomonas aeruginosa* and *Enterobacter* spp.) are the most antimicrobial-resistant bacteria that spread and cause challenges to public health in the last decade [12]. Antimicrobial resistance occurs in nature, with resistance to penicillin having been studied by Abrham and Chain in 1940, soon after Alexander Fleming discovered the drug [13]. In 1969, Swann also reported the resistance of bacteria to growth promotors and advised against their use [14]. Smith’s mathematical modelling studies showed that the use of antimicrobials in livestock farm is the major cause of the spread resistance bacteria to the environment and human health [15]. Other studies have also shown the prevalence of methicillin-resistant *staphylococcus aureus* (MARSA), which was detected more in farmers and their families compared to other populations (68.3, 14 and 0.1%, respectively [16]). The use of a non-therapeutic concentration of antimicrobials in animal feed and water was also described in Kawano’s studies as a cause of selection antimicrobial resistance in manure and environment contamination [17]. The resistance mechanism of bacteria, target alteration, drug inactivation, impermeability and efflux transfers from one organism to others via conjugation, transduction, transformation and bacteriophage systems were investigated in different studies [18]. 

A major additional cause of resistant bacteria’s increase in the environment is the non-approved use of antimicrobials in different food-producing animal chains in the last four decades. In 1930–1962, twenty classes of antimicrobials were discovered; however, since then, only a few antimicrobial classes have been approved for use in humans and animals [19]. 

**Figure 1:**
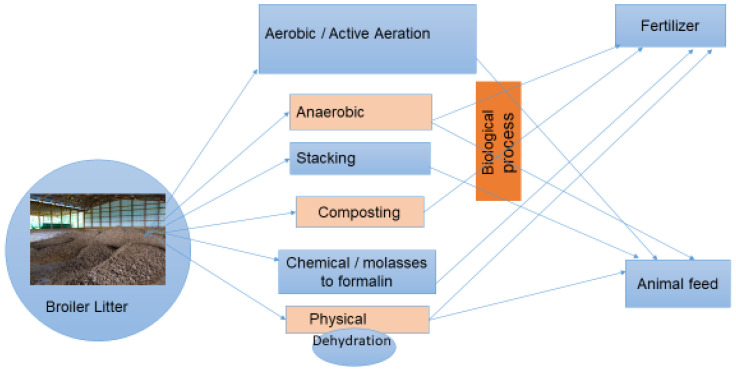
Broiler litter used as fertilizer and ruminant feed treatment options. Photo credit A.S.

To regulate the treatment processes and safe use of BL as ruminant feed, the cattle producer must vaccinate their cattle against botulism before BL use Accordingly, Israel has regulated the use of BL for feeding ruminant animals since 1971. The cheapest and most widely used BL treatment technology are microbial treatments. Since 1973, Israel regulations have required BL to be treated using a drying system at 130 °C. However, this demands more energy, and is expensive for treatment companies and farmers. Therefore, the preferred system used today are microbial treatment systems. The most popular microbial treatments of BL as animal feed in Israel are aerobic (forced aeration), anaerobic (ensiling) and stacking (Figure 1). However, conversations with ruminant producers in Israel revealed that some use an anaerobic or ensiling process alone to feed their ruminants. 

The effects of composting treatments on the reduction in pathogenic bacteria in BL were investigated in previous studies [12,20,21]. However, these studies did not explore the effects of biotic treatments used as ruminant feed. The profiles of resistant pathogenic bacterial species from conventional and organic broiler farms were examined in only a few studies, which focused on chicken organs and carcasses, and not on BL [22]. Therefore, in this study, we investigated the differences between antimicrobial-dependent and -independent farming, on the effects of treatments and their differences in terms of the reduction in four zoonotic bacteria species, Salmonella, *Escherichia coli*, *Enterococcus* and *S. aureus* in BL used as ruminant feed. 

## 2. Results

All BL samples were tested for *Enterococcus* before treatments, conducted at four locations, and the results were positive. *E. faecium* was more dominant than *E. faecalis*, as confirmed via MALDI-TOF MS. *E. coli* (11%) were also isolated before treatment in two treatment companies (R and A). The company-based treatments (R) detected only 7% of the original *Enterococcus* levels after treatment, and no *E. coli* were detected. Neither Salmonella nor *S. aureus* were detected before or after BL treatments. *E. coli* content in samples collected from windrow three days before treatment reached 26% in the southern (R) and 9% in the north-western (A) treatment companies. However, *E. coli* was not detected in the other two regions, (O) and east–west (S; antimicrobial-independent) treatment companies (Table 1). After six different BL lab-scale batch treatments (aerobic, stacking and anaerobic), none of the four pathogenic bacteria considered in this study were detected.

The largest resistance of *Enterococcus* was towards sulfafurazole and tetracycline, measured as 96 and 78%, respectively. In contrast, resistance to erythromycin, ciprofloxacin and amoxicillin was low (7–45%; Table 2). 

Sulfafurazole-resistant *Enterococcus*: sulfafurazole-resistant *Enterococcus* in 713 colonies before treatment reached levels of 96%. Of these, 94% were in a treatment company south of Israel (R), and 96% were at the other three locations (north west (A), north east (S) and other broiler farms (O) as shown in Table 2. 

**Figure 2:**
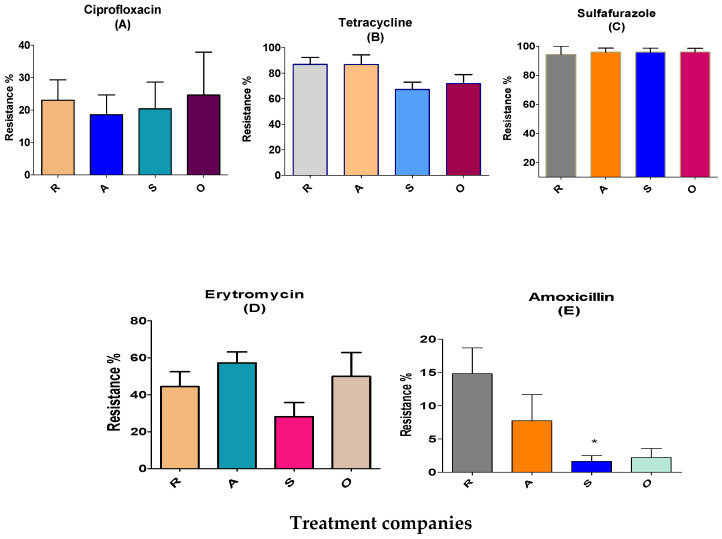
Antimicrobial resistance of *Enterococcus* isolated in BL from four different treatment companies before treatment. R, A, S (S; antimicrobial-independent) and O represent the different companies. Ciprofloxacin, 5 ppm (**A**); tetracycline, 30 ppm (**B**); sulfafurazole, 300 ppm (**C**); erythromycin, 15 ppm (**D**); and amoxicillin, 10 ppm (**E**). Means ± SEM are shown; n = 713 colonies. (* *p* < 0.05).

Tetracycline-resistant Enterococcus: 78% of the colonies were resistant to tetracycline. The distribution percentage were 87% at the R and A locations, and 67% and 72% at the S and O sites, respectively (Table 2 and Figure 2B).

Erythromycin-resistant *Enterococcus*: Erythromycin is an important antimicrobial group used in human treatments for ß-lactam resistance. The resistance of Enterococcus for erythromycin was 44%, 57%, 28% and 50% at the R, A, S (antimicrobial-independent) and O locations, respectively (Figure 2D). Ciprofloxacin resistance distribution was 19–25% at the four locations. There were few amoxicillin-resistant *Enterococcus* at antimicrobial-independent farm litters in S location (2%) but were more prevalent at the R, A and O locations (8–22%; Figure 2E). 

**Figure 3:**
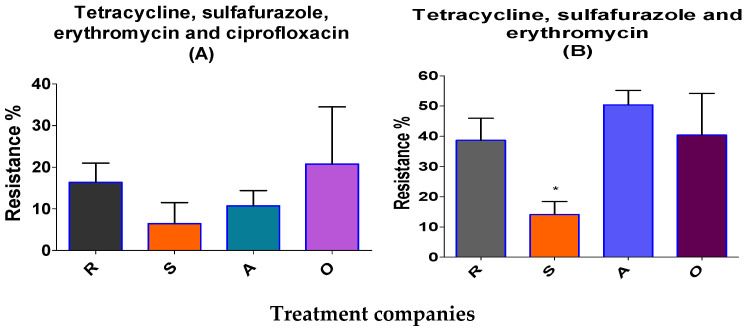
Multi-drug-resistant *Enterococcus* in broiler litter (before treatment). R, A, S (S; antimicrobial-independent) and O represent the different companies. The percentages of tetracycline-, sulfafurazole-, erythromycin-, and ciprofloxacin-resistant bacteria (**A**), and tetracycline-, sulfafurazole- and erythromycin-resistant bacteria (**B**) are presented. Means ± SEM are shown; n = 713 colonies. * *p* < 0.05).

Of the 713 isolated resistant Enterococcus colonies, 14% were multi-resistant to tetracycline, sulfafurazole and erythromycin in antimicrobial-independent farm litters; these values significantly were lower than those measured in antimicrobial-dependent broiler farm litter (38.6–50%; Figure 3B). There was a medium correlation between sulfafurazole- and tetracycline-resistant Enterococcus bacteria (r = 0.4, *p* ≤ 0.01; Table S3). Tetracycline- resistant Enterococcus also correlated with ciprofloxacin- and erythromycin-resistant bacteria at the >99.9% level of significance. Erythromycin-resistant bacteria were correlated at a medium level with ciprofloxacin-resistant bacteria (r = 0.562, *p* ≤ 0.01; Table S3).

## 3. Discussion

### 3.1. Evaluation of the Effects of Different Broiler Treatments on Antibiotic-Resistant Bacteria

The most readily detected *Enterococcus* bacteria resistant to tetracycline, amoxicillin, ciprofloxacin and erythromycin are critical priority categories of bacteria, according to the WHO (WHO, 2018. 6th revision ISBN 978-92-4-151552-8). *Enterococcus* in a healthy person does not cause infection. Indeed, these bacteria normally contribute to intestinal florae [23,24]. However, immuno-compromised hosts, especially in hospitals, can be easily infected and be afflicted by urinary tract infections, bacteraemia or endocarditis disease caused by *E. facium* [25,26]. *E. faecalis* are the most pathogenic of 50 *Enterococcus* species to humans [25]. In the current study, the most commonly Enterococcus spp. detected in BL litter by MALDI-TOF MS were *E. faecalis* and *E. faecium*. Broiler litter has the potential for generating resistant bacteria [20]. Presently, composting treatment systems for fertilizer production are more well-known than treatment systems used for preparing BL for use as ruminant feed [20,27]. Nutrition, moisture content, particle size, age of the litter, aeration condition, size of windrows and treatment systems are the most essential conditions when treating BL and eliminating pathogenic bacteria before its use as ruminant feed or fertilizer [28]. These factors were also of influence in our lab-scale treatments we used to achieve a good self-heating decompose and destroyed pathogenic microorganisms.

The biggest difference between composting treatment, aerobic, and stacking treatment processes are the duration of the treatment and the windrows aeration system. Aerobic and stacking treatments take three days and three weeks, respectively, and lead to reductions in moisture of 25–29%. The reduction in moisture is one of the critical concerns in reducing pathogenic bacteria numbers, as shown previously [29]. Biological treatment methods, namely aerobic treatment and stacking, create bio-oxidation and self-heating during treatment. This elevates the temperature gradation and reduces the moisture content. Mesophilic bacteria do not survive temperatures > 45 °C. The bio-oxidation activity releases heat, CO₂ and moisture from the treated BL into the environment. Due to thermal exposure and ammonia volatilization during the treatment process, pathogenic microorganisms are eliminated from the BL. Previous deep stacking treatment over 2–4 days and lab-scale composting treatments showed similar effects in terms of the elimination of pathogenic bacteria, as compared to the treatment performed here [20,30,31,32]. The differences in percentage of *Enteroccocus* after processing by two anaerobic treatment companies (R and S) may have been due to cross-contamination (7% and 0%, respectively). After lab-scale treatment, none of the four pathogenic bacteria were detected. 

### 3.2. Relationship between Resistant/Multi-Resistant Bacteria and Antimicrobial-Independent Therapy in Broiler Farms

The resistant Enterococcus isolated from antimicrobial-independent broiler farms (S) were significantly lower in amoxicillin-resistant *Enterococcus* (*p* < 0.05). The isolated resistant *Enterococcus* from antimicrobial-independent BL farms were also more resistant to sulfafurazole, ciprofloxacin, erythromycin and tetracycline, although these increases were not significant (*p* > 0.05). This finding agrees with those of previous studies conducted in Canada and the US that detected more resistant *E. coli* in antimicrobial-dependent farms to sulfamethoxazole, amoxicillin and tetracycline, as compared to antibiotic-using farms [15,33,34].

The multi-antimicrobial resistant *Enterococcus* analysis showed results similar to those of Kim and Subirats [20,22], who studied the subject in chicken carcasses and areas of poultry production. Multiple antimicrobial-resistant (MAR) organisms are of great concern in terms of human and animal health. Infections caused by MAR strains are more critical than are infections caused by single antimicrobial-resistant bacteria. MAR *Enterococcus* have the ability to transfer genes via plasmids and other mobile genetic elements [35,36]. *E. faecalis* correspond to those *Enterococcus* strains that are the most widely distributed in the environment [37,38]. In total, 20% of the MRA strains responsible for nosocomial infections in the US are *Enterococcus* and *Staphylococcus* strains [39,40,41]. 

The results of the current study highlight the differences between BL from antimicrobial-dependent and -independent farms in terms of three different representative antimicrobial-resistant bacteria. *E. faecium* was more readily detected than *E. fecalis*. MRA *Enterococcus* were significantly lower in independent farms’ BL for tetracycline, sulfafurazole and erythromycin (14%) resistance than the same bacteria from dependent farms (37.6–40%) were. However, when we analysed MRA *Enterococcus* for resistance to four different representative antimicrobials (tetracycline, sulfafurazole, ciprofloxacin and erythromycin), no significant difference between antimicrobial-dependent and -independent broiler farms were noted. These differences in the results confirm the unaccepted universal definition regarding multi-drug resistance (“if it is two or more classes of anti-microbials”; [42]). The occurrences of multi-drug resistance in Enterococcus were low in low antibiotic-using farms. Results from the current study thus show some similarity to those of Kim et al., 2018 and Furtula et al., 2013 on chicken carcasses and areas of poultry production [29,43]. Biotic treatments on BL at the lab scale showed a full success as regards the elimination of the four pathogenic bacteria detected. However, at the R location, 7% of *Enteroccocus* were recovered after treatments, some of which might be antimicrobial-resistant. Hence, more research is needed to conclude which treatment might be the best to eliminate the residual resistant bacteria. 

## 4. Materials and Methods

### 4.1. Chemicals and Reagents

Luria broth and Muller Hinton agar were obtained from Neogen Culture Media (Heywood, UK), saline from Sigma Aldrich (Taufkirchen, Darmstadt, Germany), and glycerol (30%) was from Bio Lab (Jerusalem, Israel). Agar medium, agar blood, MacConkey agar, Xylose, Lysine and Tergitol (XLT 4), and Kenner Fecal (KF) agar were purchased from Becton Dickinson (Bergen, NJ, USA) and Baird–Parker agar was obtained from Oxoid (Basingstoke, UK). The solutions, broth and agars were prepared at the Kimron Veterinary Institute (accredited ISO 17025). *E. coli*, *Salmonella species*, *S. aureus* and *Enterococcus species* were purchased from the Oxoid (Basingstoke, UK). The strains were used as controls during the microbiological diagnostic efforts. The American Type Culture Collection (ATCC) strain numbers for *E. coli, Salmonella species, S. aureus and Enterococcus* are 19,433, 14,028, 25,922 and 25,923, respectively. Antimicrobial disks containing amoxicillin, ciprofloxacin, tetracycline, erythromycin and sulfafurazole for resistance test were purchased from Oxoid (Basingstoke, UK).

### 4.2. Sample Collection

Most BL farmers are concentrated in the north of the country and around Jerusalem. Therefore, the largest treatment companies are found in the north-west (A, stacking treatment), north-east (S, aerobic treatment) and south Israel (R, aerobic treatment). Of the 84 batches of samples collected in 2019–2021, half were sampled before treatment by any of the three treatment companies (south (R), north west (A) and north east (S) of Israel) or broiler farms (O), and the other half was sampled after treatment by any of the three treatment companies or after lab-scale treatment. Five and four different BL batch samples were collected on different days from farms (O) and antimicrobial-independent regions (S), respectively, for aerobic, anaerobic and stacking lab-scale treatment. The other 33 batch samples were collected from the R (15), S (7), and A (11) treatment companies. Control BL samples were collected from the S region before treatment. The samples were collected from nine locations of the litter windrow utilizing a zigzag pattern. Then, the samples were placed into a cold container and transferred to the laboratory within 12 h. The samples were held at 4–8 °C pending analysis. Laboratory identification of four pathogenic bacteria was performed at the Department of Bacteriology of the Kimron Veterinary Institute, Rishon Litzion.

### 4.3. Designing a Method for Aerobic, Anaerobic and Stacking BL Treatment in the Laboratory

BL was taken from the facility over the course of a year. An airflow mechanism (aerobic treatment) containing a 22 kg BL container was attached to a Balma compressed air system (Torino, Italy) for 72 h. We supplied 4 psi air over 8 min; this was carried out three times on the first day. During the second and third days, we reduced the air amount to maintain the self-heating temperature generated by the microorganisms’ fermentation (Figure S3). The bottom of the plastic containers was drilled to release excess water during the treatment period. At the same time, anaerobic and stacking treatments were carried out in separate containers containing 12 kg of BL over the course of three weeks (Figure S4). The initial moisture contents were balanced to 40% for all treatments. The four zoonotic resistant bacteria were identified before and after all three treatments. 

### 4.4. Sample Preparation

Homogenates of BL samples (5 g) in 50 mL Falcon plastic tubes were weighed on a digital balance, and 20 glass beads and 20 mL of Luria broth (Neogen Culture Media, Lansing, MI, USA) were added. The samples were vortexed for one minute and shaken in a lab shaker for 30 min, diluted 1:10 with Luria broth and grown for 12–18 h in a 38 °C incubator (FAO, 2019. ISBN 978-92-5-131930-7). Preparation, storage and quality of the media were examined by the Food Hygiene laboratory at the Kimiron Veterinary Institute.

### 4.5. Isolation, Identification and Confirmation

After preparation, the samples were centrifuged at 200× *g* for 5 min at 4 °C. One loopful of the supernatant (0.01 mL) was taken and streaked onto to selective agar, namely MacConkey (pink/red colour; Becton Dickinson, Bergen, NJ, USA), XLT4 (black; Becton Dickinson, Bergen, NJ, USA), Baird–Parker (grey/black; Oxoid, Basingstoke, UK) and Kenner KF (purple; Becton Dickinson, Bergen, NJ, USA) agar to identify *E. coli*, *Salmonella*, *S. aureus* and *Enterococcus*, respectively. For quality control of each batch, all of the selective media were incubated at 38 °C for 24 h. As positive controls, the four bacteria were directly applied to the selective agar. The isolation and identification of pathogenic colonies were performed according to each manufacturer’s instruction manual.

Twenty isolated colonies from each selective agar were streaked onto the blood agar at 38 °C and incubated for 24 h. For Gram-positive bacteria, the incubation time was 24–36 h (FAO regional antimicrobial resistance monitoring and surveillance guidelines, 2019). The isolated colonies from the blood agar were taken for MALDI-TOF-MS analysis (Bruker, Germany) at the Bacteriology Laboratory of the Kimron Institute for confirmation of their identities [44].

### 4.6. Kirby–Bauer Test for Antimicrobial Resistance

The identities of the environmental bacteria have to be confirmed using other methods due to high false positive rates (50–70%) on selective media [45,46,47]. Therefore, the colonies were confirmed to be positive using MALDI-TOF-MS, as shown in Figure S1. The inoculation of colonies in 5 mL of saline served to match the volume of the bacterial inoculum against a 0.5 McFarland standard, thus ensuring the number of bacterial cells in each diagnostic test to be approximately the same. This was the first step in a Kirby–Bauer test used to assess antimicrobial resistance. A sterile non-toxic cotton swab was dipped into the inoculum saline buffer and rotated firmly against the upper inside wall of the tube to release excess fluid. The entire agar surface was streaked with the swab three times while turning the Mueller Hinton agar plate at 60° between each streaking. The inoculums were allowed 5 min to dry, after which time the following antimicrobial-containing disks (sulfafurazole, ciprofloxacin, amoxicillin, erythromycin or tetracycline disks; Table S1) were applied to the Hinton agar and incubated at 37 °C for 24–36 h (Figure S2). The results were interpreted as susceptibility or resistance by measuring the diameter of an inhibition zone according to the criteria stipulated by CLSI 2015 (Table S2). Negative and positive controls were included in each batch. Each isolated bacteria species was stored in Luria broth containing 25% glycerol at −80 °C for further studies. 

### 4.7. Statistical Analysis

The prevalence of *Enterococcus* resistance to five representative antimicrobials, namely, sulfafurazole, amoxicillin, ciprofloxacin, erythromycin and tetracycline, in BL was investigated using a diffusion disk test. The counts obtained were analysed using Microsoft Excel 2011 and the statistical package of Prism-GraphPad software ver. 5.04 (San Diego, CA, USA). One-way analysis of variance (ANOVA) and a Tukey multiple comparison test were used to check for any significant differences in the means of antimicrobial-resistant *Enterococcus* counts from the different locations. 

## 5. Conclusions

The source of antimicrobial-resistant bacteria is not solely due to the use of antimicrobials in livestock farming but also because resistance genes have naturally appeared for hundreds of millions of years [48], with inappropriate human use and unregulated prescriptions contributing to their increase. In Israel, from 2019, the “one health” principle was introduced in public heath institutes to reduce the resistance bacteria in the environment. However, the One Health principle has not been adequately applied to food-producing animals. In our study, resistant *Enterococcus* from antimicrobial-dependent and -independent farms showed that the low use of antimicrobial is important for reducing antimicrobial-resistant bacteria numbers in animal farms. These *Enterococcus* showing higher resistance to all antimicrobials were isolated from antimicrobial-using farms, rather than from those using low amounts of antimicrobials. Due to these reasons, untreated BL used as fertilizer and ruminant feed carries potential for environmental and public hazards. 

Most BL used as animal feed is used without treatment. Such untreated broiler litter can be a source of pathogenic bacteria, like Salmonella, that cause disease in ruminant animals and contaminate the environment. Treatment with BL eliminated human and zoonotic disease-causing pathogenic bacteria. 

## Figures and Tables

**Table 1:** Four zoonotic bacteria content in broiler litter (before treatment).

Treatment Companies	South (R)				North West (A)			
Species	*Enteroccocus*	*E. coli*	Salmonella	*Staph aureus*	*Enteroccocus*	*E. coli*	Salmonella	*Staph aureus*
N (n), C	15 (15), 283	15 (4), 39	15 (0)	15 (0)	11 (11), 153	11 (1), 34	11 (0)	11 (0)
**Treatment companies**	**North East (S; antimicrobial-independent)**				**Others (O)**			
Species	*Enteroccocus*	*E. coli*	Salmonella	*Staph aureus*	*Enteroccocus*	*E. coli*	Salmonella	*Staph aureus*
N (n), C	11 (11), 189	11 (0)	11 (0)	11 (0)	5 (5), 88	5 (0)	5 (0)	5 (0)

N; Nº of batches, n; Nº of positive batches, C; Total no of colonies for positive batches.

**Table 2:** Antibiotic-resistant bacteria in broiler litter expressed as percentage mean (before and after treatment at four representative sampling locations).

	*Enteroccocus*		*E. coli*		*Staph aureus* and Salmonella	
	Treatment		Treatment		Treatment	
	before	after	before	after	before	after
Prevalence	42/42	3/42	5/42	ND/42	ND/42	ND/42
Isolated colonies	713	42	144	ND	ND	ND
Sulfafurazole resistance, %	96	19	10	ND	ND	ND
Tetracycline resistance, %	78	16	10	ND	ND	ND
Erythromycin resistance, %	45	11	NS	NS	ND	NS
Ciprofloxacin resistance, %	22	7	5	ND	ND	ND
Amoxicillin resistance, %	7	1	7	ND	ND	ND

ND; non-detectable, NS; non sensitive.

## Data Availability

Data will be available on request from S.J.M.

## Supplementary Materials

The following supporting information can be downloaded at: https://www.mdpi.com/article/10.3390/antibiotics12071093/s1, Figure S1: Sample MALDI-TOF MS identification results described in Section 4.6; Figure S2: Streaked colony plates and antimicrobial disks in Muller Hinton medium were incubated at 38 °C described in Section 4.6; Figure S3: A compressor and drill jar for active aerobic treatment in a lab-scale study described in Section 4.3; Figure S4: Stacking and anaerobic treatment plastic bins described in Section 4.3. Table S1: Contents of representative antimicrobials in a disk diffusion test described in Section 4.6; Table S2: Zone diameters of resistant bacteria in a disk diffusion test described in Section 4.6; Table S3: Correlation between antimicrobials and resistance in Enterococcus described in Section 3.
