# Supplementary material for: Resistant Bacteria in Broiler Litter Used as Ruminant Feed: Effect of Biotic Treatment
Source: Antibiotics (Basel). 2023 Jun 23;12(7):1093. doi: 10.3390/antibiotics12071093 (PMC10376094; doi:10.3390/antibiotics12071093)
Supplement: Supplementary file 1 [file antibiotics-12-01093-s001.zip › antibiotics-2449767-supplementary.pdf]

Supplementary information

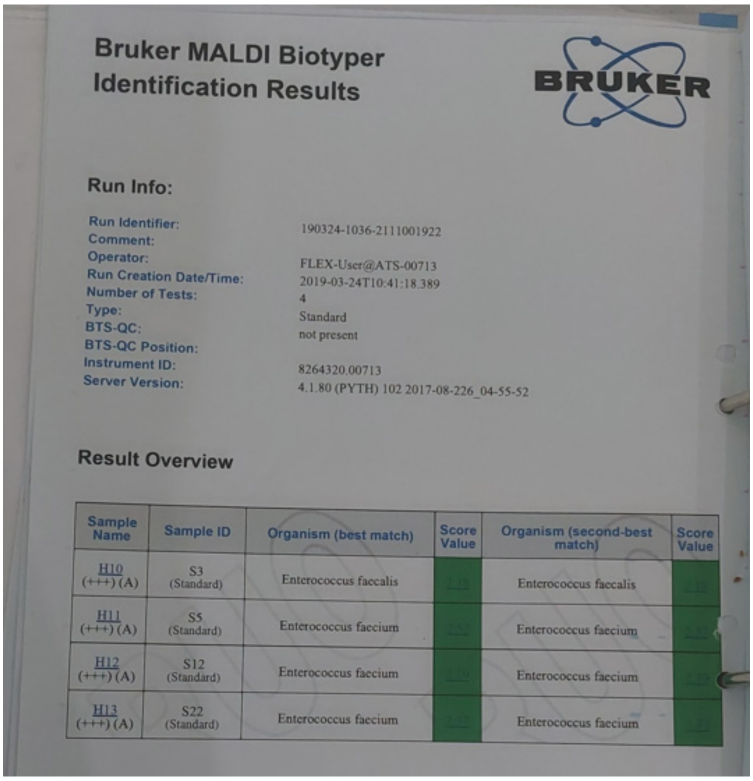

Bruker MALDI Biotyper Identification Results

BRUKER

Run Info:

Run Identifier: 190324-1036-2111001922
Comment:
Operator: FLEX-User@ATS-00713
Run Creation Date/Time: 2019-03-24T10:41:18.389
Number of Tests: 4
Type: Standard
BTS-QC: not present
BTS-QC Position:
Instrument ID: 8264320.00713
Server Version: 4.1.80 (PYTH) 102 2017-08-226_04-55-52

Result Overview

Sample Name	Sample ID	Organism (best match)	Score Value	Organism (second-best match)	Score Value
H10 (+++) (A)	S3 (Standard)	Enterococcus faecalis	[illegible]	Enterococcus faecalis	[illegible]
H11 (+++) (A)	S5 (Standard)	Enterococcus faecium	[illegible]	Enterococcus faecium	[illegible]
H12 (+++) (A)	S12 (Standard)	Enterococcus faecium	[illegible]	Enterococcus faecium	[illegible]
H13 (+++) (A)	S22 (Standard)	Enterococcus faecium	[illegible]	Enterococcus faecium	[illegible]

Figure S1. Sample MALDI-TOF MS identification results described in Section 4.6.

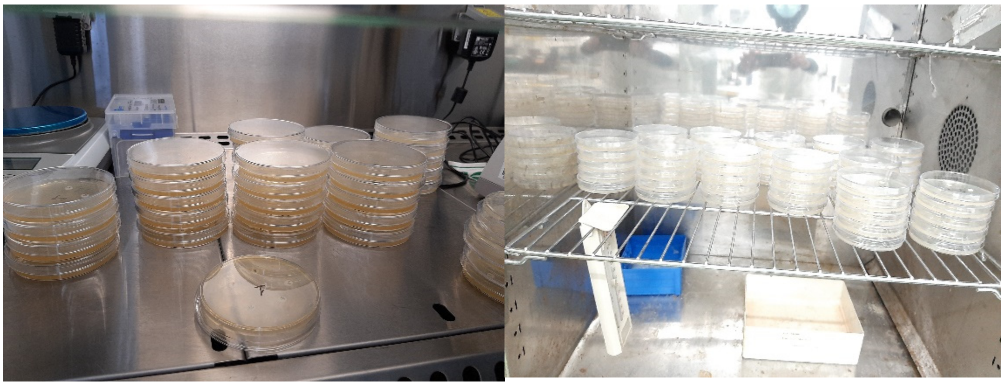

Figure S2. Streaked colony plates and anti-microbial disks in Muller Hinton medium were incubated at 38°C described in Section 4.6.

Table S1. Contents of representative anti-microbials in a disk diffusion test described in Section 4.6.

Group	Representative anti-microbials	Drug conc.	Bacteria
**ß-lactams**	amoxicillin	10ppm	Gram + and Gram -
**sulfonamides**	sulfofurazile	300ppm	Gram + and Gram -
**teteracyclines**	tetracycline	30ppm	Gram + and Gram -
**macrolides**	erythromycin	15ppm	Gram +
**fluoroquinolines**	ciprofloxacin	5ppm	Gram + and Gram -

Table S2. Zone diameters of resistant bacteria in a disk diffusion test described in Section 4.6.

Antibiotics	S. aureus ATCC 25923	Salmonella ATCC 14028	Enterococcous ATCC 29212	E. coli ATCC 25922
**amoxicillin**	≤22	≤13	≤16	≤13
**sulfofurazile**	≤12	≤12	≤22	≤12
**tetracycline**	≤14	≤11	≤14	≤11
**erythromycin**	≤13		≤13	
**ciprofloxacin**	≤15	≤20	≤15	≤15

Table S3. Correlation between anti-microbials and resistance in Enterococcus described in Section 2.

Correlations

		Sulfafurazole	*Teteracycline*	*Erythromycin*	*Ciprofloxacin*	*Amoxicillin*
Sulfafurazole	*r*	*1.000*	*0.402***	*-0.012*	*0.103*	*-0.137*
	Sig. (2-tailed)	.	*0.008*	*0.940*	*0.518*	*0.386*
	N	*42*	*42*	*42*	*42*	*42*
Teteracycline	*r*	*0.402***	*1.000*	*0.487***	*0.445***	*0.142*
	Sig. (2-tailed)	*0.008*	.	*0.001*	*0.003*	*0.370*
	N	*42*	*42*	*42*	*42*	*42*
Erythromycin	*r*	*-0.012*	*0.487***	*1.000*	*0.562***	*0.178*
	Sig. (2-tailed)	*0.940*	*0.001*	.	*0.000*	*0.260*
	N	*42*	*42*	*42*	*42*	*42*
Ciprofloxacin	*r*	*0.103*	*0.445***	*0.562***	*1.000*	*0.295*
	Sig. (2-tailed)	*0.518*	*0.003*	*0.000*	.	*0.058*
	N	*42*	*42*	*42*	*42*	*42*
Amoxicillin	*r*	*-0.137*	*0.142*	*0.178*	*0.295*	*1.000*
	Sig. (2-tailed)	*0.386*	*0.370*	*0.260*	*0.058*	.
	N	*42*	*42*	*42*	*42*	*42*

***. Correlation is significant at the 0.01 level (2-tailed).*

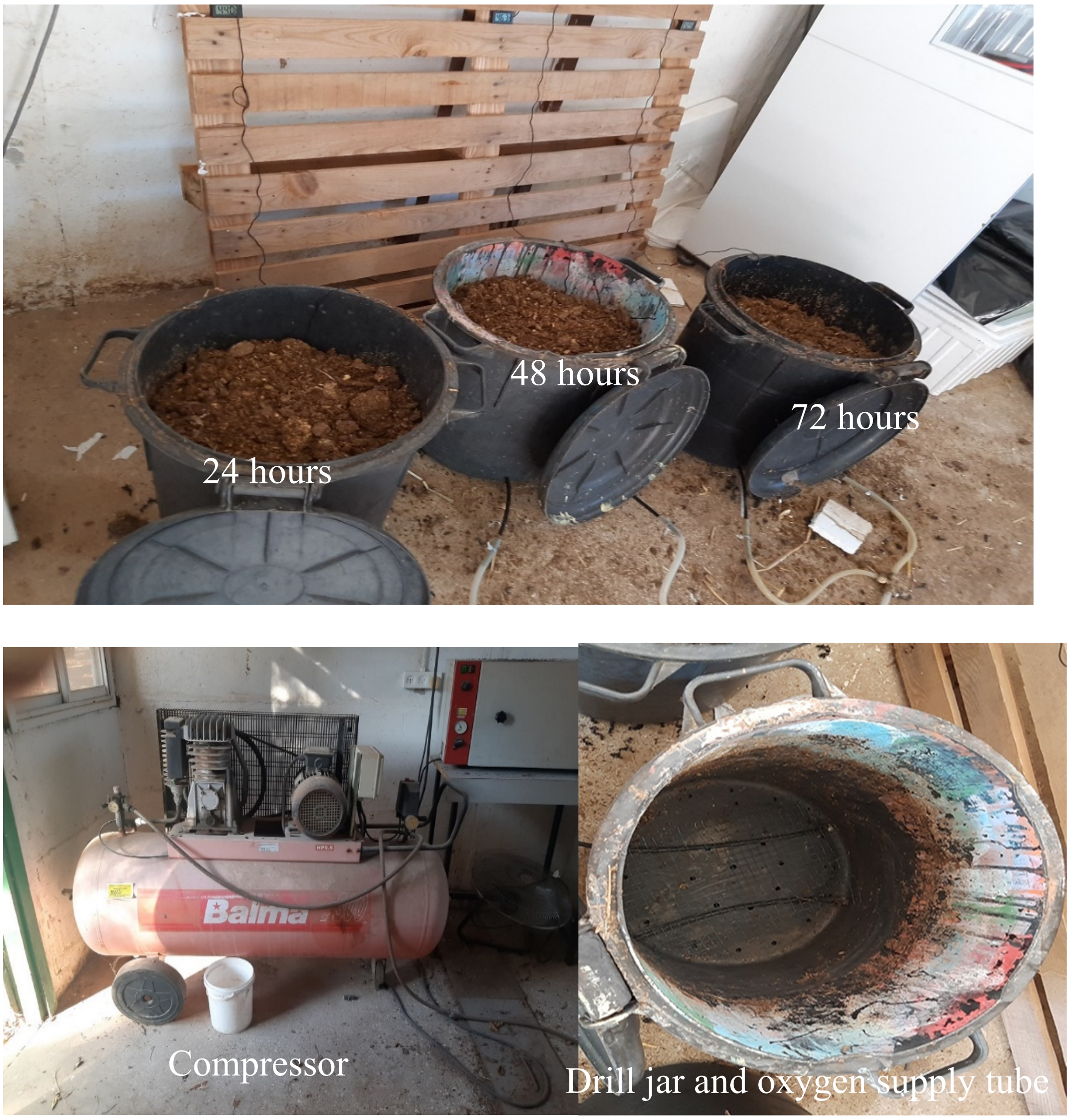

Figure S3. A compressor and drill jar for active aerobic treatment in a lab-scale study described in Section 4.3.

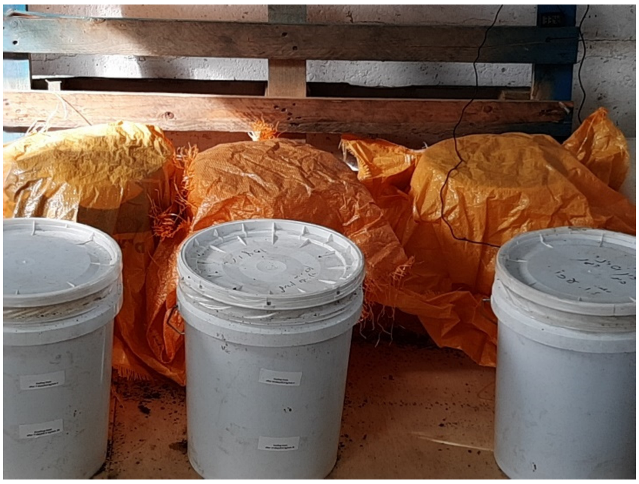

Figure S4. Stacking and anaerobic treatment plastic bins described in Section 4.3.
